# Heterogeneous CMOS Integration of InGaAs-OI nMOSFETs and Ge pMOSFETs Based on Dual-Gate Oxide Technique

**DOI:** 10.3390/mi13111806

**Published:** 2022-10-23

**Authors:** Xiaoyu Tang, Tao Hua, Yujie Liu, Zhezhe Han

**Affiliations:** 1 School of Information and Communication Engineering, Nanjing Institute of Technology, Nanjing 211167, China; 2 School of Electronic Science and Engineering, Nanjing University, Nanjing 210093, China; 3 College of Information Science and Electronic Engineering, Zhejiang University, Hangzhou 310058, China

**Keywords:** InGaAs-Insulator-Ge, MOSFET, dual-gate oxide, gate-first

## Abstract

A compatible fabrication technology for integrating InGaAs nMOSFETs and Ge pMOSFETs is developed based on the development of the two-step gate oxide fabrication strategy. The direct wafer bonding method was utilized to obtain the InGaAs-Insulator-Ge structure, providing the heterogeneous channels for CMOS integration. Superior transistor characteristics were achieved by optimizing the InGaAs gate oxide with a self-cleaning process in atomic layer deposition, and modifying the Ge gate oxide by the ozone post oxidation (OPO) technique, in the sequential two-step gate oxide fabrication process. With the combination of the gate-first fabrication process, superior on- and off-state characteristics, i.e., on current up to 8.3 µA/μm and leakage as low as 10^−^^6^ µA/μm, have been demonstrated in the integrated MOSFETs, together with the preferable symmetric output characteristics that promises excellent CMOS performances.

## 1. Introduction

In spite of the attractive benefits in the continuous scaling down of silicon (Si) metal-oxide-semiconductor field-effect-transistors (MOSFETs), it is increasingly difficult to go further due to problems such as short channel effects, quantum effects and so on. Many innovative methods are proposed with novel channel materials, device architectures and physical mechanisms [1,2,3,4]. As promising alternative channel materials, Germanium and III–V materials have attracted lots of research interest [5,6]. Germanium (Ge) has both higher electron mobility and higher hole mobility than Si. However, the fabrication of Germanium nMOSFET is difficult due to the metal-related gap states, which could induce a Fermi-level pinning effect [7]. Comparatively, Ge pMOSFETs has demonstrated its high-channel-mobility advantages with the application of a high-pressure oxidized GeO_2_ or HfO_2_/Al_2_O_3_ stack based on the plasma after oxidation treatment as the gate oxide [8,9]. On the other hand, InGaAs with high electron mobility is an attractive candidate for nMOSFET, where, for example, a high-performance gate-all-around (GAA) InGaAs MOSFETs has been fabricated using ion implantation-based ohmic source/drain with saturation current of 1.17 mA/μm [10].

Correspondingly, in the continuous evolution of the CMOS technology, heterogeneous integration between InGaAs nMOSFETs and Ge pMOSFETs is attracting growing research interest, with the expectation to deliver advantageous nMOSFETs and pMOSFETs performance simultaneously [11,12,13,14]. However, the integrated fabrication of these two MOSFETs has long been challenging, considering the different fabrication and processing methodology of the gate oxide. For the oxide/semiconductor interface engineering, fabrication procedure of Ge-based pMOSFETs usually utilizes post-oxidation treatment to suppress the interface traps in Ge gate oxide [8], while for InGaAs-based nMOSFETs, the self-cleaning effect before oxide deposition in atomic layer deposition (ALD) is preferred [15,16]. Meanwhile, the thermal budget during the fabrication of the S/D terminal with the dopant activation in the well could also introduce severe degradation of the gate stack quality [10]. Although a metal source/drain (S/D) has been proposed in ultra-shallow junctions formation with low contact resistances based on low temperature [17], the possible influences on the integrated two gate stacks of Ge- and InGaAs-based MOSFETs are still to be evaluated.

In this work, the dual-gate stack fabrication method that combines but individually optimizes the InGaAs gate oxide and Ge gate oxide was developed to construct the integrated p- and n-MOSFETs. By capping InGaAs channel using Al_2_O_3_ film while operating the ozone post oxidation (OPO) treatment to Ge channel, which barely utilizes an additional lithography step, the gate oxide fabrication and modification on the two channel materials was accomplished. Together with application of the gate-first fabrication strategy, which was selected based on the direct comparison between the gate-first and gate-last processing strategies, integrated InGaAs-OI nMOSFETs and Ge pMOSFETs with symmetric output characteristics and well reduced off-state leakage have been achieved, paving a way to the future high-performance CMOS.

## 2. Experiments

**Figure 1:**
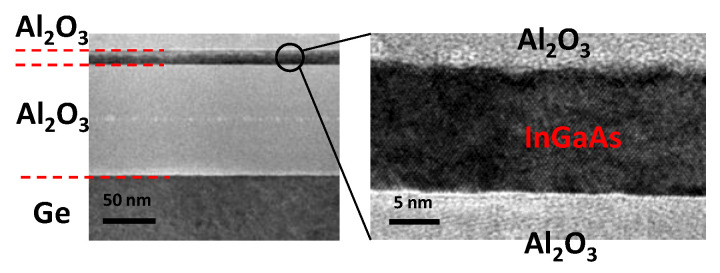
TEM cross section of the InGaAs (8 nm)/Al_2_O_3_ (100 nm)/Ge structure.

To prepare the InGaAs-Insulator-Ge substrate, (100) n-Ge wafer (resistivity: 10~20 ohm.cm) and p-In_0.53_Ga_0.47_As/InP wafer (doping: ~10^16^ cm^−3^) was manually bonded together in the air, with the 50 nm-thick Al_2_O_3_ film deposited by atomic layer deposition (ALD) on both wafers [18]. After annealing process in N_2_ ambient at 300 °C for 30 min, InP was selectively etched by HCl solution to fabricate the InGaAs 100 nm-thick Al_2_O_3_-Ge substrate. The cross-sectional TEM observation of the final structure in Figure 1 shows that an 8 nm-thick InGaAs layer has been successfully transferred to the top of 100 nm-thick Al_2_O_3_ on the Ge substrate with sharp interface and excellent uniformity.

**Figure 2:**
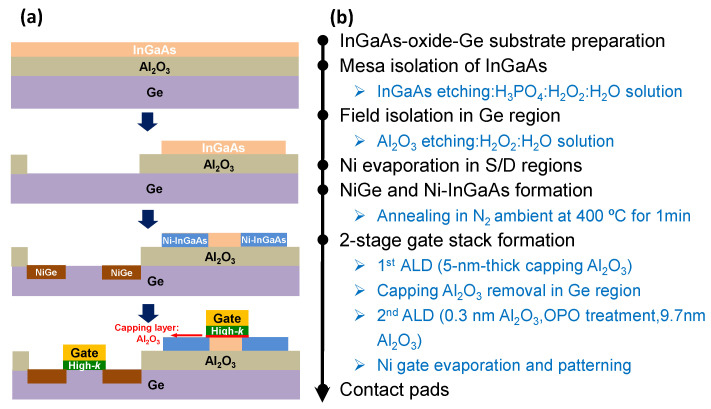
(**a**) Cross section views of devices and (**b**) key fabrication process of gate-last fabrication scheme of InGaAs-OI nMOSFET and Ge pMOSFET with dual-gate oxide technique.

The gate-last process has been firstly conducted and explored, where the metal S/D were produced before the fabrication of the gate stack, with the advantages of maximum elimination of the thermal budget on the gate terminal. Figure 2 summarizes the gate-last fabrication scheme of the InGaAs-OI nMOSFETs and Ge pMOSFETs using the InGaAs-Al_2_O_3_-Ge substrate above. The active area of the InGaAs was defined by etching the InGaAs island pattern using H_3_PO_4_:H_2_O_2_:H_2_O solution while Ge active area was patterned and exposed by removing the buried oxide (BOX: 100 nm Al_2_O_3_). A Ni-InGaAs and NiGe metalized alloy, formed by electron beam evaporation and a subsequent rapid thermal annealing in N_2_ ambiemt at 400 °C for 1 min, was introduced to construct the metal Source/Drain (S/D) structure for both InGaAs-OI nMOSFETs and Ge pMOSFETs at the same time. Then, the substrate cleaning was performed in the acetone, de-ionized water and HCl solution, which was followed by the gate oxide deposition in ALD at 300 °C with trimethylaluminum (TMA) and H_2_O as precursors, producing a 5 nm-thick Al_2_O_3_ layer on the InGaAs and Ge surfaces. Subsequently, the 5 nm-thick Al_2_O_3_ in the gate region of Ge MOSFETs was etched by BHF solution to prepare for the following specific gate dielectric fabrication, while the 5 nm-thick Al_2_O_3_ on the InGaAs MOSFETs was preserved to protect the oxide/InGaAs interface. Then, 0.3 nm-thick Al_2_O_3_ was deposited on Ge and InGaAs surfaces, which was followed by an in situ ozone post oxidation (OPO) treatment for 1 min in the 10% O_3_/O_2_ ambient with the pressure of ~100 Pa. The interlayer of GeO_x_ was formed in OPO treatment with modified interface quality of Ge gate stack. Thereafter, a 9.7 nm-thick Al_2_O_3_ layer was deposited immediately to obtain a 10 nm-thick Al_2_O_3_/Ge stack, which also leads to a gate oxide thickness of 15 nm in the InGaAs MOSFETs. All of the Al_2_O_3_ deposition and OPO treatment in ALD chamber were performed at 300 °C, with the consideration of alleviating the thermal budget on the metal S/D. Finally, Ni electrodes were fabricated in gate and S/D terminals for the electrical contact.

## 3. Results and Discussion

**Figure 3:**
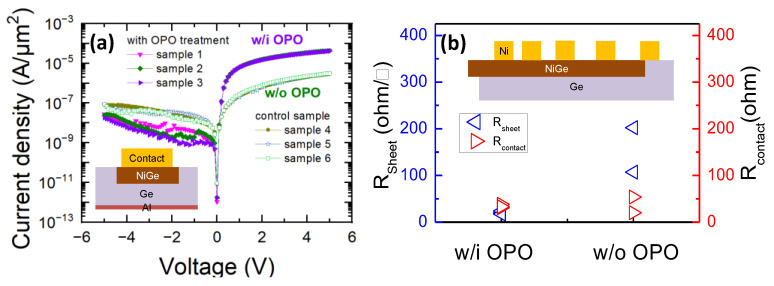
(**a**) Current characteristics of NiGe/Ge junctions with OPO treatment and control sample, (**b**) contact resistance and film resistance of NiGe films with OPO treatment and control sample.

As illustrated in Figure 2, the OPO treatment has been employed as a novel strategy for the interface engineering of Al_2_O_3_/Ge for low interface state density and small EOT [8]. Considering this, the OPO treatment after the NiGe/Ge junctions could impact on the previously fabricated metal S/D, NiGe/Ge junctions with additional OPO treatment subsequent to metallization as well as control sample with no OPO treatment were fabricated. The fabrication of the NiGe/Ge junctions began with the series cleaning of the n-Ge wafers by acetone, de-ionized water and BHF solution. A total of 20 nm Ni was then evaporated by electron beam evaporation and annealed in N_2_ ambient at 400 °C for 1 min to produce the shallow alloyed junction. Then, the OPO treatment at 300 °C for 1 min in ALD was performed on some of the samples, while the others experienced no OPO treatment. Ultimately, Ni was evaporated as the contact pad, while Al was deposited as the back contact. Figure 3a shows the current density of NiGe/Ge junctions with and without OPO treatment. The on-current of NiGe/Ge junctions with OPO treatment is one order larger than those control samples. Figure 3b gives the correspondingly calculated sheet resistance and contact resistance of NiGe/Ge structure. It could be observed the sheet resistance can be reduced by 80% for NiGe film with OPO treatment, while the contact resistance in both cases has similar values. That is, the OPO treatment in the gate stack process is generally beneficial for the metal S/D in the Ge pMOSFETs with lowered sheet resistance.

**Figure 4:**
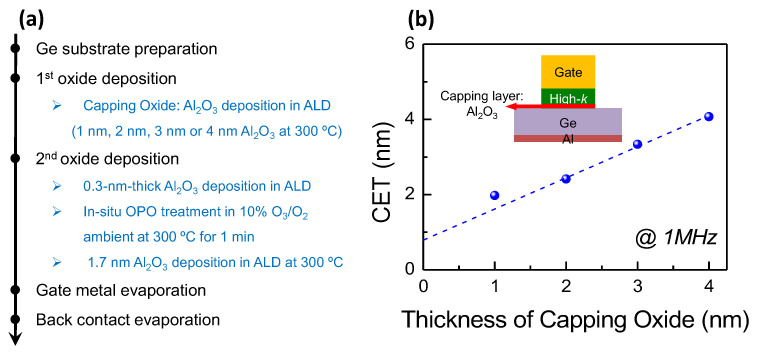
Experiment of Ge MOSCAPs with different capping oxide thickness from 1 nm to 4 nm. (**a**) Fabrication process, (**b**) capacitance equivalent thicknesses of different MOSCAPs.

Meanwhile, to evaluate the effectiveness of the 5 nm-thick Al_2_O_3_ in protecting the oxide/InGaAs interface from the influence of the OPO treatment, experiments of Ni/Al_2_O_3_/Ge/Al MOSCAPs with different capping Al_2_O_3_ in OPO treatment were constructed, as shown in Figure 4a. Al_2_O_3_ of 1 nm, 2 nm, 3 nm or 4 nm thicknesses were deposited on pre-cleaned Ge substrates as capping oxide films. The in situ OPO treatment was carried out on the stack after 0.3 nm-thick Al_2_O_3_ deposited, followed by another 1.7 nm-thick Al_2_O_3_ deposition. Ni and Al were evaporated as top and back contacts. The capacitance equivalent thicknesses (CET) of Ni/Al_2_O_3_/Ge/Al MOSCAPs are presented in Figure 4b. It could be determined, based on the relationship between CET and Al_2_O_3_ thickness that, 2 nm-thick Al_2_O_3_ is sufficient to alleviate the OPO effect on channel surface. Therefore, considering the 5 nm-thick Al_2_O_3_ as utilized on the InGaAs channel, the OPO treatment influence could be neglected.

**Figure 5:**
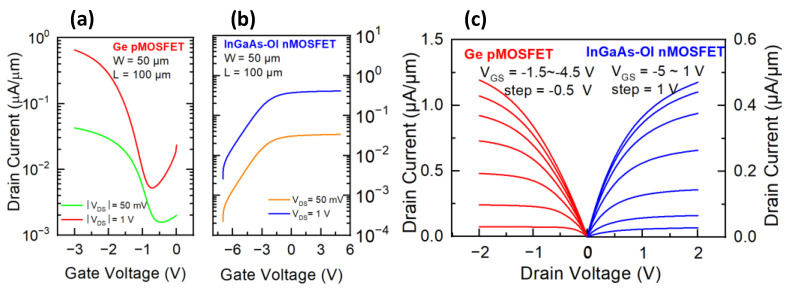
Electron characteristics of Ge pMOSFET and InGaAs-OI nMOSFET with width/length = 50 μm/50 μm under gate-last fabrication process (**a**) I_d_-V_g_ curves of Ge pMOSFET, (**b**) I_d_-V_g_ curves of InGaAs-OI nMOSFET, (**c**) I_d_-V_d_ curves of both devices.

The corresponding transfer and output characteristics of the gate-last InGaAs-OI nMOSFETs and Ge pMOSFETs (width/length = 50 μm/50 μm) were characterized to directly examine the potentials of the integration of the two MOSFETs in Figure 5. Generally, the two devices show symmetric output performance with current level reaching ~1 μA/μm. However, the leakage current in both p- and n-MOSFET in level of 10^−3^ μA/μm is relatively higher. Considering that, the gate stack has been well protected from the thermal budget during the fabrication of the metal S/D in the gate last process, the device leakage characteristics could be a result of the exposure of the oxide/semiconductor interface to the prior S/D fabrication process, which could introduce detrimental contaminations.

**Figure 6:**
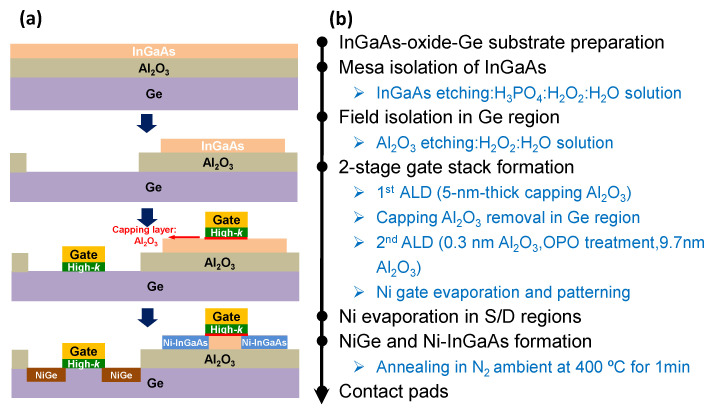
(**a**) Cross section views of devices and (**b**) Key fabrication process of gate-first fabrication scheme of InGaAs-OI nMOSFET and Ge pMOSFET with dual-gate oxide technique.

**Figure 7:**
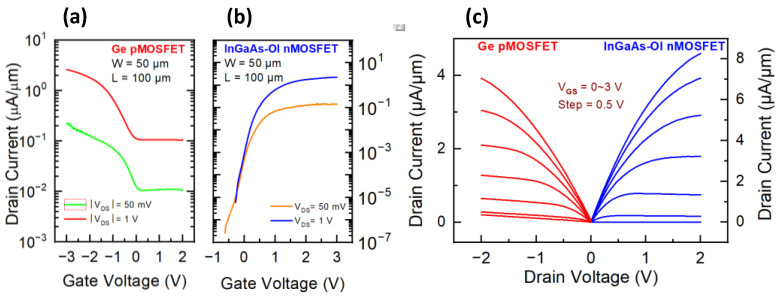
Electron characteristics of Ge pMOSFET and InGaAs-OI nMOSFET with width/length = 50 μm/100 μm under gate-first fabrication process (**a**) I_d_-V_g_ curves of Ge pMOSFET, (**b**) I_d_-V_g_ curves of InGaAs-OI nMOSFET, (**c**) I_d_-V_d_ curves of both devices.

Correspondingly, gate-first process utilizing similar dual-gate oxide method, i.e., two-step oxidation technique to enable the OPO treatment in the Ge MOSFETs while protecting the oxide/InGasAs interface, was proposed and demonstrated as illustrated in Figure 6, where both the gate oxide deposition and processing were conducted before the fabrication of the metal S/D. Electrical performances of InGaAs-OI nMOSFET and Ge pMOSFET with geometries of width/length = 50 μm/100 μm are shown in Figure 7. The on/off ratio of InGaAs-OI nMOSFET has reached 10^6^. The on-state current of Ge pMOSFET could reach 3.91 µA/µm, which is in the same level with the on-state current of InGaAs-OI nMOSFET as 8.3 µA/µm, promising for superior CMOS operation. The apparently suppressed off-state leakage current in the InGaAs-OI nMOSFET indicate that the semiconductor/dielectric interface protection via the gate first process is especially effective in the InGaAs MOSFET.

**Figure 8:**
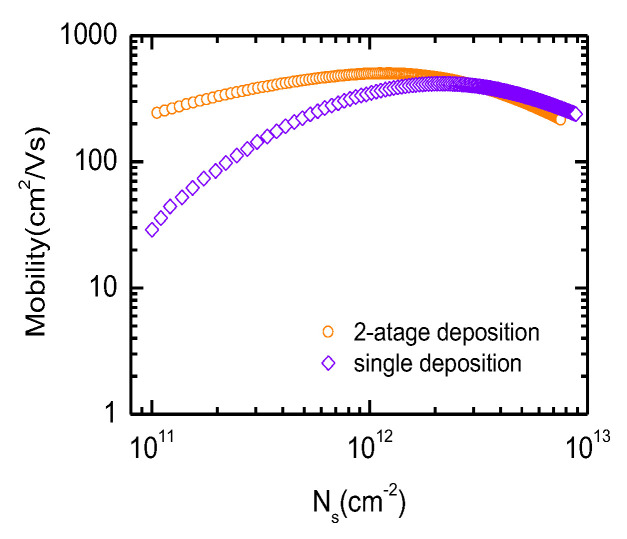
Electron mobility comparison of InGaAs-OI nMOSFET with two-stage gate oxide and one-stage gate oxide (width/length = 50 μm/100 μm).

The influence of the OPO treatment and the post fabrication of the metal S/D on the carrier mobility of the InGaAs MOSFET has been further validated. Electron mobility of InGaAs-OI nMOSFETs fabricated with different gate stacks were extracted by split-CV measurement based on the I_d_-V_g_ characteristics @ V_g_ = 10 mV and C_gc_-V_g_ characteristics at frequency of 50 kHz, as shown in Figure 8. The peak mobility of InGaAs-OI nMOSFETs with (5 + 20) nm-thick Al_2_O_3_ has been calculated to be 501 cm^2^/V·s. This value is even higher than the 416 cm^2^/V·s from the device with directly deposited 25 nm-thick Al_2_O_3_. Therefore, the gate-first process-based dual-gate-oxide approach is generally more beneficial for the integration of InGaAs-OI nMOSFET and Ge pMOSFET technique approach.

## 4. Conclusions

In this work, the dual-gate oxide fabrication and processing technique has been developed for the heterogeneous CMOS integration of InGaAs-OI nMOSFETs and Ge pMOSFETs. With a two-step oxidation technique to enable the OPO treatment in the Ge MOSFETs while protecting the oxide/InGasAs interface as self-cleaned in the ALD before deposition, excellent device on-state current with symmetric output characteristics and well suppressed off-state leakage has been achieved. This work provides a promising fabrication approach to the future CMOS technology with integrated high-electron-mobility InGaAs-OI-based nMOSFETs and high-hole-mobility Ge pMOSFETs.
